# FOXP3 is a promising and potential candidate gene in generalised vitiligo susceptibility

**DOI:** 10.3389/fgene.2015.00249

**Published:** 2015-07-22

**Authors:** Parveen Jahan, Surekha Tippisetty, Prasanna L. Komaravalli

**Affiliations:** ^1^Department of Zoology, School of Sciences, Maulana Azad National Urdu UniversityHyderabad, India; ^2^Department of Genetics, University College of Sciences, Osmania UniversityHyderabad, India

**Keywords:** autoimmune, forkhead box P3, immunoregulatory gene, polymorphisms, susceptibility, vitiligo

Vitiligo, the very frequent ancient idiopathic dermatological disorder is characterized by chronic and progressive autoimmune loss of melanocytes from the basal layers of the epidermis that results in white macules on the affected skin. Although non-contagious, the phenotype of this disorder has high impact on the psychological behavior of the affected individuals than their physical ability; more often lead to social isolation. Over the past decade, the prevalence of vitiligo is on rise, ranges from 0.5 to 2% worldwide, whereas 0.09 to 8.0% in India (Lu et al., 2007; Shah et al., 2008; Krüger and Schallreuter, 2012). Because of the intricacy of the disease, although there is a wide range of treatment modalities available, none of the therapeutic options are effective enough to thwart further progression or complete cure from depigmentation.

This complex inflammatory disorder involves a combination of pathogenic effects of multiple susceptible loci and unknown environmental triggers which may act independently or together in the destruction of melanocytes of the affected area (Le Poole et al., 1993a). Several hypotheses have been put forward to explain the etio-pathogenesis of this multi-factorial disfiguring disorder, but are not concrete enough to substantiate the underlying mechanism(s) involved in the disease development. To recognize genes that mediate in vitiligo susceptibility, diverse approaches such as gene expression analyses, candidate gene association, genome-wide linkage, and genome wide association studies have been exploited (Allam and Riad, 2013).

Various studies reported that the initial triggering event of vitiligo is oxidative stress; however, inflammation and autoimmunity act as major contributors in the pathogenesis, progression and severity of the disease (Jain et al., 2011; Deo et al., 2013; Laddha et al., 2013; Spritz, 2013). The autoimmune loss of pigmentation is allied with infiltrates of T cells and macrophages, and the progression of the disease is due to the decreased CD4^+^/CD8^+^ cell ratio which is cytotoxic to melanocytes (Lili et al., 2012). Animal studies (h3TA2) have further established the fact that depletion of regulatory T (Treg) cells in knockout mice up regulate the proinflammatory cytokines and mediate the progressive depigmentation; nevertheless adoptive transfer of Treg cells induce vitiligo remission in treated mice (Chatterjee et al., 2014).

Treg cells are a unique CD4^+^ T lymphocyte lineage that plays an indispensable role in maintaining immunological unresponsiveness to self-antigens and in suppressing excessive immune responses that are detrimental to the host. Defects in the number and function of Treg cells in the peripheral blood and lesional skin accentuate the role of Treg cells in vitiligo pathogenesis (Dwivedi et al., 2015). The crucial master transcription factor that regulates the fate and identity of Treg cells which play an imperative role in preventing autoimmunity is forkhead box protein 3 (FOXP3). This key immunoregulatory *FOXP3* gene belongs to forkhead/winged-helix transcription factor family, located at Xp11.23 in humans is 1296 bp in length and consists of 11 translated exons that encode a protein with 431 amino-acids. It contains a proline rich N-terminal repressor domain that suppresses the expression of target genes, a zinc-finger (amino acid 200–223), and leucine-zipper motif (amino acids 240–261) that allow FOXP3 homo- or hetero dimerization and a conserved DNA binding forkhead domain (amino acid 338–421).

*FOXP3* gene is vital for the development, proliferation, and function of CD4^+^CD25^+^ Treg cells and therefore essential for the maintenance of immune homeostasis (Zheng and Rudensky, 2007; Zheng et al., 2007). Alongside the Treg cells development, *FOXP3* gene also has a role in the expression of cytokines and regulation of other T cell subsets through NF-AT/NF-κB and Smad7 pathways (Song et al., 2012). Approximately 700 *FOXP3* bound genes are up/down regulated in FOXP3^+^ T cells, suggesting that FOXP3 acts as both transcriptional activator and repressor. The functional or quantitative modification of FOXP3 leads to lack of efficient CD4^+^CD25^+^Treg cells, resulting in immune dysregulation, polyendocrinopathy, enteropathy, X-linked syndrome (Bennett et al., 2001; Wildin et al., 2002), scurfy mice and other autoimmune diseases including vitiligo supports the importance of Treg cells and FOXP3 in keeping autoreactive T cells in check (Yagi et al., 2004; Lahl et al., 2007).

Comprehensive reanalysis of genome wide data of 33 biological candidate genes previously implicated in generalized vitiligo (ACE, AIRE, CAT, CD4, CLEC11A, COMT, CTLA4, C12orf10, DDR1, EDN1, ESR1, FAS, FBXO11, FOXD3, FOXP3, GSTM1, GSTT1, IL1RN, IL10, KITLG, MBL2, NFE2L2, PDGFRA-KIT, PTGS2, STAT4, TAP1-PSMB8, TGFBR2, TNF, TSLP, TXNDC5, UVRAG, VDR, and XBP1) has identified three candidate genes XBP1, FOXP3, and TSLP in association with vitiligo that are not shared by other autoimmune diseases (Chen et al., 2005; Birlea et al., 2010, 2011; Jin et al., 2010; Tang et al., 2013). A meta-analysis conducted by Birlea et al. (2010) screening 37 SNPs of FOXP3 gene found greatest significance with a promoter SNP rs3761547 and strong linkage disequilibrium with rs11798415 and rs5906843 block; moreover, rs11798415 retained as a strongest marker than other SNPs (rs11798415, rs5906843, rs5906777, and rs4824755) in this region. No further studies are available on FOXP3 gene polymorphisms in relation to vitiligo, however, in psoriatic patients, one of the upstream SNPs of FOXP3 gene rs3761548 was found significant association with the disease (Shen et al., 2010). The study demonstrated the functional significance of rs3761548 AA genotype which causes the loss of binding with transcription factors, E47 and C-Myb, leading to defective transcription of FOXP3. Moreover, the A allele of this polymorphism is associated with a dramatic reduction in luciferase activity compared with the C allele (Shen et al., 2010). Later rs3761548 AA genotype was confirmed to be the lowest FOXP3 producing genotype and suggested its role in fewer Treg cells and/or weaker immune suppressive function in other autoimmune diseases (Wu et al., 2012).

To further evaluate the *FOXP3* gene polymorphisms and their involvement in vitiligo pathogenesis, recent studies from Indian and Han Chinese populations screened three promoter SNPs of *FOXP3* gene (rs3761548, rs2232365, and rs5902434) and found significant association of rs3761548 and rs2232365 with vitiligo risk (Jahan et al., 2013; Song et al., 2013). The rs2232365 polymorphism located within the putative DNA-binding site of other transcription factor GATA-3, directly regulates FOXP3 expression and controls Treg cell function via interaction with the regulatory regions of the FOXP3 locus. The GG genotype of rs2232365 decreases the FOXP3 expression and affects Treg cell function, thus disrupt the Th1/Th2 balance, leading to the pro-inflammatory disease vitiligo (Song et al., 2012; Wu et al., 2012).

Cell based investigations conducted in vitiligo evaluating quantitative and qualitative analysis of Treg cells in lesional/perilesional and blood samples had provided mixed results. According to Zhou et al. (2012) no variation was observed in systemic Treg cell population between vitiligo patients and controls. In contrary, it was suggested that the reduced cell number and impaired function of natural Treg cells is associated with imbalance of CD4^+^/CD8^+^ ratio and was suggested to be involved in the T-cell mediated pathogenesis of vitiligo and its progression (Klarquist et al., 2010; Lili et al., 2012; Dwivedi et al., 2013). At large, most of the investigators implicated the imbalance in Treg cells in the disease pathogenesis.

Though evidences are mounting toward the involvement of FOXP3 functional SNPs and quantitative and qualitative variation of CD4^+^CD25^+^Treg cells individually pointing toward their role in the diseases pathogenesis, studies are lacking to correlate the *FOXP3* gene variants and Treg cell behavior in vitiligo. It is well established that FOXP3 is a master transcription factor which influences the quality and quantity of Treg cell population thus the immune homeostasis of the host. Keeping in view the pivotal role of Treg cells in the maintenance of immune balance and association of its master controller *FOXP3* gene with autoimmune diseases, in our opinion, *FOXP3* is a potential candidate gene which should be screened thoroughly for known and novel functional genetic variants and epigenetic modifications that regulate its expression; further to correlate the impact of these variations on Treg cell development, stability and function will be promising in understanding the genetic heterogeneity of vitiligo. Establishing the role of *FOXP3* gene through family based studies in various ethnic groups will help in delineating the genetic and non-genetic contributing factors that are imperative for this multifactorial disorder and also in understanding the population based variation in its incidence. Moreover, the interventions targeting Treg cells/*FOXP3* gene networks that could suppress the immune responses may open new insights for effective therapeutic approaches for vitiligo.

## Conflict of interest statement

The authors declare that the research was conducted in the absence of any commercial or financial relationships that could be construed as a potential conflict of interest.
